# Genetisch modifizierte regulatorische T-Zellen: Therapiekonzepte und ihr regulatorischer Rahmen

**DOI:** 10.1007/s00103-020-03230-8

**Published:** 2020-10-16

**Authors:** Attila Sebe, Brigitte Anliker, Juliane Rau, Matthias Renner

**Affiliations:** Abteilung Medizinische Biotechnologie, Paul-Ehrlich-Institut, Paul-Ehrlich-Str. 51–59, 63225 Langen, Deutschland

**Keywords:** Regulatorische T‑Zellen, Immuntoleranz, Genetisch modifizierte Zellen, Adoptive T‑Zelltherapie, Regulatorische Rahmenbedingungen, Regulatory T cells, Immune tolerance, Genetically modified cells, Adoptive T‑cell therapy, Regulatory framework

## Abstract

Adoptive T‑Zelltherapien sind neuartige Konzepte zur Behandlung verschiedener Krankheiten. CAR-T-Zellen sind dabei als Letztlinientherapie für fortgeschrittene B‑Zelllymphome und die B‑Zellleukämie etabliert und zugelassen. TCR-basierte T‑Zellen als Behandlungsoption verschiedener hämatologischer und solider Tumoren befinden sich in der klinischen Entwicklung. Genetisch modifizierte regulatorische T‑Zellen stehen dagegen noch am Anfang ihrer klinischen Entwicklung zur Induktion von Immuntoleranz in einer Vielzahl von Anwendungsgebieten.

In diesem Artikel wird zunächst ein Überblick über die Funktion der regulatorischen T‑Zellen für die Induktion der Immuntoleranz sowie über ihre Rolle im Pathomechanismus bestimmter Immunerkrankungen gegeben und der aktuelle Stand der klinischen Entwicklungen von therapeutischen Ansätzen auf Basis genetisch modifizierter regulatorischer T‑Zellen zusammengefasst. Im Weiteren werden die regulatorisch-wissenschaftlichen Anforderungen und Herausforderungen hinsichtlich Herstellung und Qualitätskontrolle sowie nichtklinischer und klinischer Testung genetisch modifizierter regulatorischer T‑Zellen als Arzneimittel für neuartige Therapien diskutiert.

## Abstract (en)

Adoptive T‑cell therapies are emerging tools to combat various human diseases. CAR‑T cells are approved and marketed as last line therapeutics in advanced B‑cell lymphomas and leukemias. TCR-engineered T cells are being evaluated in clinical trials for a variety of hematological and solid tumors. Genetically modified regulatory T cells, however, are still in the initial stages of clinical development for the induction of immune tolerance in various indications.

Here we outline the general role of regulatory T cells in establishing self-tolerance and the mechanisms by which these suppress the effector immune cells. Further, the role of regulatory T cells in the pathomechanism of certain immune diseases is presented, and the current status of clinical developments of genetically modified Treg cells is discussed. We also present the regulatory framework for genetically modified regulatory T cells as advanced therapy medicinal products, including aspects of manufacture and quality control, as well as nonclinical and clinical development requirements.

## Einleitung

Das adaptive Immunsystem hat die Aufgabe, eindringende Krankheitserreger zu erkennen und zu zerstören. In Verbindung mit dem angeborenen Immunsystem vermittelt es auch die allogene Transplantatabstoßung über zelluläre und humorale Mechanismen. Eigengewebe wird durch das adaptive Immunsystem aufgrund existierender Immuntoleranzmechanismen nicht angegriffen.

Die Immunantwort ist im Regelfall durch ein homöostatisches Gleichgewicht zwischen dem regulatorischen Suppressor- und dem Effektorarm gekennzeichnet. Versagt die Suppression zur Steuerung des Effektorarms, gerät das Immunsystem aus dem Gleichgewicht und es entstehen Autoimmun- und Entzündungskrankheiten [1]. Die derzeitig verfügbaren therapeutischen Konzepte zur Bekämpfung dieser Krankheiten beschränken sich auf den Einsatz unspezifischer Immunsuppressiva [2]. Der zunehmende Erkenntnisgewinn zur Funktionsweise dieses Gleichgewichts zwischen Regulator- und Effektorarm erlaubt die Entwicklung neuer biologischer Arzneimittel zur Bekämpfung dieser Krankheiten.

Die Immuntoleranz wird durch komplementäre rezessive und dominante Mechanismen aufrechterhalten. Rezessive Immuntoleranzmechanismen laufen innerhalb einer Zelle ab. Dazu gehört die Zerstörung selbstreaktiver Immunzellen sowie eine Erhöhung der Anzahl inhibitorischer Rezeptoren auf diesen Zellen, um die Schwelle für ihre Aktivierung zu heben. Im Gegensatz dazu sind dominante Immuntoleranzmechanismen zellextrinsisch und werden von Untergruppen spezialisierter Immunzellen, wie den regulatorischen T‑Zellen (Tregs) ausgeführt. Tregs sind in der Lage, die Aktivierung, Expansion und Funktion anderer Immunzellen aktiv zu unterdrücken, und sind die einzigen Zellen, die ausschließlich der Induktion und Aufrechterhaltung der Immuntoleranz dienen [2].

Etwa 5–7 % aller CD4+-T-Zellen im menschlichen Körper sind regulatorische T‑Zellen. Die Identität und Funktion von Tregs werden durch die Expression verschiedener Proteine in diesen Zellen charakterisiert. Dazu gehören, neben dem zytotoxischen T‑Lymphozyten-Protein 4 (CTL4-A), der Interleukin(IL)-2-Rezeptor-Untereinheit‑α (CD25) und dem die Transkription aufrechterhaltenden Faktor STAT5 [2], insbesondere das Forkhead-Box-Protein P3 (FOXP3). Die Schlüsselfunktion von FOXP3 für die Aufrechterhaltung der Immuntoleranz durch die regulatorischen T‑Zellen wird durch FOXP3-Genmutationen deutlich. FOXP3-defiziente Mäuse entwickeln ein autoinflammatorisches Syndrom in mehreren Organen [3, 4], beim Menschen sind FOXP3-Mutationen ursächlich für das immun-dysregulations-polyendokrinopathie-enteropathie-X-chromosomale (IPEX-)Syndrom, das in den ersten Lebensjahren bei Knaben auftritt und eine Knochenmarktransplantation erfordert [5, 6].

Man unterscheidet zwei Arten von Treg-Zellen, t‑Tregs und p‑Tregs, basierend auf ihrem Ursprung im Thymus oder in der Peripherie. Das T‑Zellrezeptorrepertoire der beiden Zellpopulationen ist unterschiedlich [7]. Die t‑Treg-Zellen bilden eine stabile Population von Suppressorzellen mit einer Häufung von T‑Zellantigenrezeptoren (TCRs), welche Selbstantigene erkennen. P‑Tregs werden in der Peripherie aus herkömmlichen CD4+-T-Zellen, die mit Antigenen, insbesondere im Darm, in Kontakt kamen und durch hohe Mengen an transformierendem Wachstumsfaktor‑β (TGFβ) und Retinsäure stimuliert wurden, erzeugt [8]. Das TCR-Repertoire für p‑Treg-Zellen umfasst auch TCRs, die *nicht selbst* Antigene aus Viren oder kommensalen Mikroorganismen, die für die Aufrechterhaltung der Schleimhauttoleranz wichtig sind, erkennen [9]. Derzeit ist kein Marker bekannt, mit dem sich beim Menschen t‑Treg-Zellen von p‑Treg-Zellen unterscheiden lassen. Daher sind Treg-Zellen, die zu therapeutischen Zwecken aus peripherem Blut isoliert wurden, wahrscheinlich eine Kombination von t‑Treg-Zellen und p‑Treg-Zellen.

Treg-Zellen vermitteln die Immuntoleranz über verschiedene Mechanismen [10]. Sie exprimieren entzündungshemmende Mediatoren wie IL-10, TGFβ und IL-35 und reduzieren den IL-2-Spiegel. Darüber hinaus exprimieren sie negativ regulatorische Zelloberflächenrezeptoren wie CTLA‑4, CD39 und CD73, wodurch sie T‑Zellen direkt oder indirekt durch Modulation von antigenpräsentierenden Zellen ansprechen. Zusätzlich führt die Bindung von Tregs an antigenpräsentierende Zellen zur Trogozytose, wodurch die Antigenpräsentation verringert wird. Treg-Zellen können durch die immunsuppressiven Proteine auch das Mikromilieu im Gewebe verändern. Dadurch proliferieren weitere immunsuppressive Zellpopulationen wie Treg-Zellen mit unterschiedlichen Spezifitäten und T‑regulatorische Tr1-Zellen [11].

### Tregs-assoziierte Erkrankungen

Zahlreiche Autoimmunkrankheiten haben ihre Ursachen in einem Mangel an funktionellen Treg-Zellen oder in einer Mutation in den für die Treg-Funktion essenziellen Proteinen. Der Mangel an STAT5b resultiert in einem Immundefekt [12, 13], Mutationen im CTLA4-Protein in der Entwicklung eines autosomal dominanten Immundysregulationssyndroms [14]. Durch eine verminderte Expression von FOXP3 in Treg-Zellen von Patienten mit Myasthenia gravis werden autoreaktive T‑Zellen weniger gehemmt, die charakteristischen Symptome dieser Muskelschwächekrankheit verschlimmern sich [15]. Bei Patienten mit systemischem Lupus erythematodes ist der Anteil aktivierter Treg-Zellen in den frühen Krankheitsphasen verringert [16] und die Funktion der vorhandenen Treg-Zellen durch eine geringe Menge an phosphorylierten STAT5 beeinträchtigt [17]. Patienten mit Lupusnephritis und antineutrophiler zytoplasmatischer antikörperassoziierter Vaskulitis haben ebenfalls geringere Level an Treg-Zellen in den Nieren [18]. Ebenso ist in den frühen Phasen des Typ-1-Diabetes die Anzahl der Treg-Zellen signifikant verringert [19]. In Psoriasispatienten ist die Zahl an CCR5 exprimierenden Treg-Zellen reduziert, ebenso sind die Funktion und die chemotaktischen Eigenschaften der Zellen eingeschränkt [20]. Eine beeinträchtigte regulatorische Funktion der Tregs wurde auch bei Patienten mit multipler Sklerose beobachtet, was letztendlich zum Verlust der immunologischen Selbsttoleranz führt [21].

Die Rolle von Treg-Zellen ist bei Organtransplantationen und bei Tumoren weitaus komplexer einzuschätzen. Normalerweise proliferieren Tregs während der aktiven Immunreaktion auf das Transplantat und infiltrieren das transplantierte Organ allmählich. In den frühen Phasen nach Transplantation können die Treg-Zellen die alloimmune Entzündungsreaktion jedoch noch nicht unterdrücken [22]. Bei Tumorpatienten ist die Zahl der Treg-Zellen im Blut und auch im Tumorgewebe erhöht. Die Rekrutierung von Tregs in den Tumor wird durch Chemokine vermittelt, die im Tumormikromilieu produziert werden. Tregs unterdrücken dort die Antitumorimmunität, indem sie ein Eliminieren der Tumorzellen durch antigenspezifische CD8+-T-Zellen hemmen [23]. Auch scheinen die Tumorzellen die intratumorale Retention und das Überleben der Treg-Zellen zu begünstigen [24]. Daher wird die intratumorale Akkumulation von Treg-Zellen als Marker für einen metastasierten Tumor im fortgeschrittenen Stadium und für eine verringerte Überlebensrate bei Krebspatienten diskutiert [25].

Mit diesem Wissen wurden in den letzten Jahren Strategien entwickelt, um Treg-Zellen therapeutisch zu nutzen. Allerdings gibt es noch keine spezifischen Arzneimittel, die die Treg-Aktivität positiv oder negativ beeinflussen können. IL‑2 kann zwar die Treg-Zellfunktion verbessern, es fördert aber allerdings auch die Funktion der Effektor-T-Zellen (Teffs). Da Treg-Zellen eine höhere Affinität zu IL‑2 und eine stärkere IL-2-rezeptorvermittelte Signalübertragung aufweisen als Teff-Zellen, wurde Patienten in klinischen Studien nur niedrig dosiertes IL‑2 verabreicht. Eine Verbesserung der klinischen Symptomatik wurde bei Patienten mit Hepatitis-C-Virus(HCV)-induzierter Vaskulitis [26] und steroidresistenter Graft-versus-Host Disease (GvHD) beobachtet [27]. Die Identifikation eines sicheren und spezifischen therapeutischen Dosisfensters gestaltet sich allerdings schwierig, da dieses stark von patienten- und indikationsspezifischen Parametern abhängt und eine suboptimale Dosis zur Stimulation von Effektor-T-Zellen führen würde.

Ein alternativer Ansatz zur Treg-Beeinflussung mittels IL‑2 ist die Gabe autologer ex vivo expandierter Treg-Zellen. Mit diesem zelltherapeutischen Ansatz wurden bereits vielversprechende Ergebnisse bei Patienten mit chronischer GvHD [28] und Typ-1-Diabetes [29] erzielt. Die ex vivo expandierten polyklonalen Zellen zeigten jedoch eine limitierte Lebensdauer und ihre Funktion war durch den Mangel an Spezifität eingeschränkt. Zielführender wäre hier sicherlich die Verwendung antigenspezifischer Treg-Zellen [30].

Bei Krebspatienten wurde im Gegensatz dazu die Depletion von Treg-Zellen versucht. Hierbei besteht jedoch die Gefahr von schweren Nebenwirkungen durch Autoimmuneffekte [31]. Mit NRP1, einem in intratumoralen Tregs selektiv und stark exprimierten Rezeptor für den Liganden SEMA4A, wurde ein potenzielles Ziel identifiziert, die immunsupprimierende Funktion intratumoraler Tregs aufzuheben. Damit könnte die Wirksamkeit von Immuncheckpointinhibitoren verbessert werden, während, anders als bei der Treg-Depletion, die periphere Immuntoleranz unverändert ist [32].

Die jüngsten Entwicklungen erlauben die Expression von klonalen, spezifischen rekombinanten TCRs oder chimären Antigenrezeptoren (CARs) in Treg-Zellen nach Transfer von Nukleinsäuren, die für diese Proteine kodieren, um die Spezifizität der Tregs gegen die entsprechenden Antigene im betroffenen Gewebe umzuleiten. Durch CRISPR-Cas9-mediiertes Genom-Editing ließen sich gleichzeitig mehrere Gene in den Tregs spezifisch editieren oder HLA-defiziente Tregs designen, um allogene Treg-Zellen „off-the-shelf“ für verschiedene klinische Anwendungen zur Verfügung zu stellen. Mit derartigen Strategien ließen sich Treg-Zellen mit verbesserter Funktionalität und schnellerer Verfügbarkeit für therapeutische Ansätze bei Organtransplantationen und zur Behandlung von Autoimmunerkrankungen oder Krebs generieren.

Im Folgenden werden die regulatorisch-wissenschaftlichen Anforderungen und Herausforderungen hinsichtlich Herstellung und Qualitätskontrolle sowie nichtklinischer und klinischer Testung genetisch modifizierter regulatorischer T‑Zellen als Arzneimittel für neuartige Therapien diskutiert.

## Herstellung und Qualitätskontrolle regulatorischer T-Zellen

Die Herstellung von genetisch modifizierten Treg-Zellen unterliegt einem komplexen Prozess. Zunächst müssen mononukleäre Zellen (PBMC) durch Apherese aus peripherem Blut als zelluläres Ausgangsmaterial gewonnen werden. Die Entnahme auch von autologen Zellen eines Patienten unterliegt den nationalen Vorgaben für die Spendertestung in Anlehnung an die europäische Richtlinie 2002/98/EG. Darüber hinaus benötigen die Entnahmezentren eine Herstellungserlaubnis nach § 13 Arzneimittelgesetz (AMG) für die Gewinnung der Zellen. Da der Anteil von Tregs im peripheren Blut äußerst gering ist, muss eine Anreicherung der Zellen durch verschiedene Selektionsschritte erfolgen. Dazu werden in der Regel zunächst CD8+-T-Lymphozyten und teilweise auch B‑Lymphozyten durch Negativselektion mithilfe von magnetischen Beads abgereichert und dann die Treg-Zielzellen über Anti-CD4-, Anti-CD25- und Anti-CD45RA-Antikörper, die charakteristische Oberflächenantigene für die Identität der Treg-Zellen darstellen, angereichert. Diese Anreicherung erfolgt mittlerweile häufig mithilfe von fluoreszenzaktivierter Zellselektion (FACS), was eine besondere Herausforderung für die Durchführung unter Reinraumbedingungen darstellt, sofern nicht mittlerweile verfügbare Geräte mit geschlossenem System verwendet werden. Durch FACS ist auch eine Negativselektion über Anti-CD127 möglich.

Die Gewinnung einer möglichst reinen Treg-Zellpopulation ist essenziell, um in den nachfolgenden Herstellungsschritten, wie Zellaktivierung und genetische Modifikation, möglichst wenige konventionelle T‑Zellen zu aktivieren, zu modifizieren bzw. zu expandieren, um damit einerseits negative Effekte auf die Qualität des Treg-Zellprodukts zu minimieren, aber auch um höchstmögliche Sicherheit und Effizienz der Therapie nach Applikation des Zellprodukts zu gewährleisten. Verunreinigungen durch T‑Effektorzellen, die wie das eigentliche Treg-Zellprodukt z. B. einen alloreaktiven rekombinanten CAR exprimieren, würden durch ihre Aktivierung in vivo den eigentlichen therapeutischen Effekt der Immunsuppression nach Transplantation ins Gegenteil verkehren und eine massive Abstoßungsreaktion gegenüber dem Transplantat hervorrufen.

Nach abgeschlossener Selektion erfolgt eine Aktivierung der Tregs, die häufig mit CD3- und CD28-Antikörper-Beads sowie über IL-2-Gabe durchgeführt wird [33]. Als Alternative wird die Aktivierung über letal bestrahlte, rekombinant antigenpräsentierende Zellen beschrieben. Diese Zellen exprimieren nach genetischer Modifikation CD86 und CD64. Nach Beladung von CD86 mit einem CD3-spezifischen Antikörper erfolgt eine entsprechende Stimulation der Treg-Zellen [34, 35]. Die Aktivierung soll sehr effizient erfolgen [36], allerdings wird die Produktion des Arzneimittels deutlich komplexer, da sowohl die vollständig letale Bestrahlung dieser Zellen als auch ihre ausreichende Eliminierung sichergestellt werden müssen. Letzteres gilt zwar auch für die Verwendung der Antikörper-Beads, insgesamt ist das mit ihrer Verwendung assoziierte Risikopotenzial für den Patienten allerdings als geringer einzuschätzen. Durch eine zusätzliche Gabe von Rapamycin lässt sich die selektive Depletion von T‑Effektorzellen erreichen und damit eine Instabilität der Tregs durch Expansion der T‑Effektorzellen verhindern [33].

Die genetische Modifikation von Treg-Zellen kann auf verschiedenen Wegen erfolgen, einerseits durch ungerichteten Transfer der genetischen Sequenz, z. B. durch Transduktion der Zellen mithilfe von retro- oder lentiviralen Vektoren. Soll eine zielgerichtete Mutation oder Deletion z. B. des HLA-Lokus erfolgen, kann durch Einsatz der CRISPR/Cas9-Technik der entsprechende Sequenzbereich spezifisch genomeditiert werden. In allen Fällen wird das Werkzeug für den Ex-vivo-Gentransfer, sei es viraler Vektor, Plasmid, mRNA oder Protein, zumindest als Ausgangsmaterial klassifiziert. Demzufolge haben die Herstellung und Qualitätskontrolle entsprechenden Anforderungen zu genügen.

Nach genetischer Modifikation kann eine weitere Expansion der Zellen z. B. in Gegenwart von Anti-CD3/Anti-CD28-Antikörper-Beads und IL‑2 erfolgen, bis die gewünschte Zellzahl erreicht ist. Nach Abreicherung prozessbedingter Verunreinigungen wie Antikörper-Beads, Materialien zur Transduktion oder Mediumkomponenten werden die Zellen formuliert, abgefüllt und entweder kryokonserviert oder zur direkten Applikation an den Patienten freigegeben. Bei direkter Anwendung liegen jedoch nicht alle Ergebnisse aus den geforderten Freigabetests vor Zertifizierung der Charge und ihrer Anwendung vor. Daher muss durch geeignete In-Prozess-Testung und erweiterte Produktcharakterisierung das Risiko für den Patienten durch die fehlenden Daten minimiert werden. Aus regulatorischer Sicht ist die Kryokonservierung einer direkten Anwendung immer vorzuziehen, soweit kritische Qualitätsparameter wie Viabilität und Aktivität der Tregs nach Auftauen dieses zulassen.

Die Freigabespezifikationen variieren je nach Art des finalen Produkts. Generell sind Tests auf Sterilität, Endotoxin- und Mykoplasmenfreiheit durchzuführen sowie die Parameter Identität, Gehalt, also Zellzahl und Vitalität der Zellen zu prüfen. Die Identität kann durch den kombinierten Nachweis von positiver oder hoher Expression von CD4, CD25 und CD45RA erfolgen [37]. Weitere Identitätsmarker in Abhängigkeit von den gewünschten zellulären Treg-Subtypen können der Nachweis hoher FOXP3-Expression bzw. geringer oder fehlender Expression von CD127 sein. Eine weitergehende phänotypische und funktionelle Analyse möglicher Treg-Subtypen ist im Rahmen der Charakterisierung des Zellprodukts und auch für eine Optimierung des Herstellungsprozesses in jedem Fall sinnvoll. Ein besonderes Augenmerk ist auf produkt- und prozessbedingte Verunreinigungen zu legen und dabei insbesondere auf noch vorhandene konventionelle T‑Zellen und je nach Therapiekonzept v. a. auf konventionelle genetisch modifizierte T‑Zellen, da diese den eigentlichen therapeutischen Ansatz konterkarieren und damit das Nutzen-Risiko-Verhältnis der Therapie deutlich verschlechtern können. Je nach Verfahren der genetischen Modifikation sind Analysen zu residualen Vektorpartikeln oder Werkzeugen für die Genomeditierung notwendig. Die biologische Aktivität des Produkts lässt sich, vor allem in der frühen klinischen Entwicklung zumindest in Näherung über die Zahl der genetisch modifizierten Zellen, also die Modifikationseffizienz sowie die Expression des eingebrachten Gens, soweit dies im therapeutischen Konzept vorgesehen ist, abbilden.

Da derzeit genetisch modifizierte regulatorische T‑Zellen noch in der sehr frühen klinischen Entwicklung stecken, mit weltweit nur wenigen laufenden klinischen Prüfungen, sind auch die regulatorischen Vorgaben hinsichtlich Herstellung, Qualitätskontrolle und Qualität der jeweiligen Produkte und Prüfpräparate bei Weitem nicht fixiert. Daher ist die kontinuierliche regulatorische Interaktion zwischen den Entwicklern und den regulatorischen Behörden unentbehrlich für ein rasches und erfolgreiches Voranbringen dieses vielversprechenden Therapieansatzes.

## Therapiekonzepte auf Basis regulatorischer T-Zellen

Auch wenn derzeit verschiedene therapeutische Ansätze auf Basis von regulatorischen T‑Zellen diskutiert werden, befindet sich der überwiegende Teil noch in der frühen Entwicklungsphase und wird, wenn überhaupt schon, derzeit noch in präklinischen In-vitro-Studien und in Tiermodellen getestet.

Das IPEX-Syndrom ist eine autoimmunologische Erkrankung basierend auf Mutationen im FOXP3-Gen, die zum Funktionsverlust dieses wichtigen Transkriptionsfaktors und damit zu nichtfunktionellen Tregs führen [6]. Bei dieser und anderen monogenetischen Erkrankungen kann eine klassische Gentherapie zum Einsatz kommen, bei der eine funktionelle Kopie des FOXP3-Gens in autologe T‑Zellen eingebracht wird. Denkbar ist, beispielsweise mithilfe eines integrierenden Vektors, die korrekte Version der FOXP3-cDNA ex vivo in CD4+-T-Lymphozyten der betroffenen Patienten einzubringen, die transduzierten T‑Lymphozyten zu expandieren und diese im Anschluss dem Patienten zu reinfundieren. Dieser Ansatz wurde in Mäusen mit einer induzierten GvHD bereits erfolgreich getestet [38]. Ebenso konnte für die Therapie des IPEX-Syndroms die Wirksamkeit der Tregs in nichtklinischen Studien gezeigt werden [39–41]. Eine erste klinische Prüfung ist seit Mai 2019 angekündigt [42], aber bisher in keinem Register für klinische Prüfungen gelistet.

Besonders wichtig für die klinische Anwendung ist der präklinische Nachweis, dass in vivo stabile FOXP3-Expressionslevel erreicht werden und die Expression unter inflammatorischen Bedingungen nicht verloren geht.

### Rekombinante TCR- und CAR-modifizierte Tregs

Neben der Substitution mit der korrekten Sequenz sind gentherapeutische Konzepte in der Entwicklung, bei denen Tregs mit einem zusätzlichen spezifischen rekombinanten TCR oder CAR ausgestattet werden, um bei unerwünschten immunologischen Reaktionen die Antigenspezifität der Tregs zu steuern [43]. Ziel des Ansatzes ist es, die Balance zwischen den Teff- und den Treg-Zellen, die das Zielantigen erkennen, wiederherzustellen bzw. die Konzentration der spezifischen Tregs soweit zu erhöhen, dass eine Toleranz gegen das Zielantigen induziert wird. Denkbar ist dieses Konzept bei autoimmunologischen Erkrankungen wie Typ-1-Diabetes, aber auch bei allogenen Organtransplantationen. Dass TCR- und CAR-modifizierte Tregs tatsächlich die Immunreaktion gegen körpereigenes Gewebe oder das transplantierte Organ verhindern können, wurde bisher in entsprechenden Tiermodellen gezeigt [44–46]. Ob und wie gut diese Tregs im Menschen funktionieren, ist derzeit noch unklar. Im November letzten Jahres wurde mit der Genehmigung der ersten klinischen Prüfung mit CAR-Tregs in Großbritannien (EudraCT Nr. 2019-001730-34, EU Clinical Trials Register) eine wichtige Hürde in Richtung klinischer Anwendung genommen. Bei dieser klinischen Prüfung der Phase I/IIa soll getestet werden, ob autologe Tregs, die mit einem gegen HLA-A2-gerichteten CAR ausgestattet wurden, bei einer Nierentransplantation von einem HLA-A2-positiven Spender auf einen HLA-A2-negativen Empfänger die Abstoßungsreaktion des Empfängers gegen das Transplantat vermindern können. Erwartet wird dabei, dass die HLA-A2-CAR-Tregs nach der Reinfusion in den Empfänger an das HLA-A2-Antigen im transplantierten Organ binden und so zur Aktivierung und Proliferation angeregt werden. Durch die starke Präsenz aktivierter Tregs wiederum soll die proinflammatorische Immunreaktion, welche aufgrund der HLA-A2-Diskrepanz durch die T‑Effektorzellen des Empfängers hervorgerufen wird und normalerweise zur Abstoßung des Transplantats führen würde, soweit unterbunden werden, dass die starke immunsuppressive Standardtherapie reduziert bzw. im Idealfall ganz gestoppt werden kann. Auch bei diesem Ansatz ist es essenziell, dass die genetisch modifizierten CAR-Tregs ihren Phänotyp in vivo beibehalten und sich dieser nicht, beispielsweise durch den Verlust der FOXP3–Expression, zum Phänotyp einer T‑Effektorzelle hin ändert. Dieses wäre fatal, da diese Zellen durch den HLA-A2-CAR die besten Voraussetzungen für eine gezielte und potente immunologische Reaktion gegen das Transplantat hätten. Ähnlich kritisch ist eine mögliche Kontamination des finalen Produkts mit HLA-A2-T-Effektorzellen zu sehen, die während der Herstellung allerdings unerwünscht mit dem CAR-kodierenden Vektor transduziert wurden. Das Risiko solcher Verunreinigungen sollte in nichtklinischen Spiking-Experimenten analysiert werden. Dabei wird das Zellprodukt bis zu einem bestimmten Prozentsatz mit genetisch modifizierten T‑Effektorzellen verunreinigt, um zu prüfen, ob dadurch im Tier GvHD oder anderweitige immunologische Reaktionen induziert werden können und ob die CAR-rekombinanten Tregs die Proliferation der T‑Effektorzellen kontrollieren können.

TCR- und CAR-modifizierte Tregs können auch bei anderen Indikationen zum Einsatz kommen, um z. B. unerwünschte Arzneimittelwirkungen im Sinne von Anti-Drug-Antikörpern entgegenzuwirken. Ein Beispiel hierfür ist die Inhibitorbildung bei Patienten mit Hämophilie, die mit Gerinnungsfaktoren substituiert werden [47]. Die X‑chromosomal-rezessiv vererbbare Hämophilie A tritt bei Männern mit einer Häufigkeit von etwa 1:5000 auf. Etwa ein Drittel der substituierten Patienten mit schwerer Hämophilie A entwickelt neutralisierende Antikörper gegen Faktor VIII [48]; die Inzidenz in Studien mit vorher unbehandelten Patienten wurde mit bis zu 52 % angegeben [49]. In In-vitro- und teilweise auch In-vivo-Studien konnte gezeigt werden, dass TCR- oder CAR-modifizierte Tregs prophylaktisch und bei bestehenden Inhibitoren eine Immunantwort unterdrücken können [50, 51]. Dabei wurde nicht nur nachgewiesen, dass Faktor-VIII-spezifische CAR-Tregs Faktor-VIII-spezifische Teffs supprimieren können, sondern auch, dass Faktor-VIII-spezifische B‑Zellen unterdrückt werden können. Die genaue Wirkweise, d. h., ob die genetisch modifizierten Tregs eher die B‑Gedächtniszellen und/oder die T‑Helferzellen unterdrücken, ist derzeit aber noch unklar. Zum Zeitpunkt der Publikation dieser Daten war die Immuntoleranzinduktion die einzige verfügbare Therapieoption [48]. Mit der Zulassung von Emicizumab in der EU und in der Schweiz 2018 steht mittlerweile eine Routineprophylaxe für Patienten aller Altersgruppen, die eine Hämophilie A mit Inhibitoren haben, zur Verfügung [52]. Als weiterer Therapieansatz für Hämophiliepatienten mit Inhibitoren wird auf Basis vielversprechender nichtklinischer Daten die AAV-basierte Gentherapie diskutiert [53]. Der Bedarf an einer Immuntoleranzinduktion ist dadurch nicht verschwunden [54]; die relative Häufigkeit der zu therapierenden seltenen Erkrankung und das Vorhandensein von Therapiealternativen werden jedoch Einfluss auf das Design und die Durchführbarkeit der ersten klinischen Prüfung(en) haben.

## Nichtklinische Anforderungen

### Nachweis der Spezifität, Funktionalität und Persistenz

Auch wenn die Anwendungsgebiete genetisch modifizierter Tregs sehr unterschiedlich sein können, einige Fragestellungen und Herausforderungen bei der nichtklinischen Entwicklung haben alle diese Ansätze gemein.

Zum einen muss die Spezifität des Ansatzes überprüft werden. Dieses kann bei TCR- oder CAR-modifizierten T‑Zellen durch Co-Kultivierung mit antigenexprimierenden Zielzellen erfolgen. Durch die spezifische Interaktion zwischen dem rekombinanten TCR bzw. CAR und dem Zielantigen wird die T‑Zelle aktiviert, was in Zellkultur durch die Expression bestimmter Markerproteine und die nachfolgende Proliferation der T‑Zellen messbar ist. In Kontrollansätzen mit Zielzellen ohne Zielantigenexpression oder nichtmodifizierten T‑Zellen sollten keine Aktivierung und Proliferation der T‑Zellen erkennbar sein, was die Spezifität des Ansatzes belegen würde.

Zum anderen ist zu zeigen, dass die genetisch modifizierte T‑Zelle die von ihr erwartete Funktion nach Aktivierung durch das Zielantigen ausführt. Bei onkologischen Ansätzen mit TCR- oder CAR-T-Effektorzellen erfolgt dieses üblicherweise durch den Nachweis der Zytotoxizität gegenüber den Zielzellen. Bei TCR- oder CAR-modifizierten Tregs ist ein funktioneller Nachweis komplexer und erfolgt z. B. durch die Suppression von Teff-Zellen. Dazu müssen neben den Zielzellen und den TCR- oder CAR-Tregs auch entsprechende T‑Effektorzellen verwendet werden; das prozentuale Verhältnis zwischen den Teff-Zellen und Tregs wird dabei variiert, sodass die Suppression der Teff-Zellen durch die Tregs anhand der graduellen Inhibierung der Zytotoxizität der Teff-Zellen erkennbar wird. Anstelle der Zytotoxizität können aber auch andere Parameter wie die Sekretion inflammatorischer Zytokine durch die T‑Effektorzellen oder ihre Proliferation herangezogen werden.

Ein wesentliches Kriterium für die Anwendung von genetisch modifizierten Tregs ist die Persistenz der Zellen in vivo. Für eine erfolgreiche Therapie sollten die genetisch modifizierten Tregs über einen längeren Zeitraum überleben sowie ihre Aktivität und ihren Treg-Phänotyp beibehalten. Ein entsprechender Nachweis dafür ist aber oftmals schwierig, da Tregs von bestimmten Zytokinen, insbesondere von IL‑2, abhängig sind. Zum Überleben der Zellen, insbesondere zum längerfristigen Überleben in einem heterologen Tiermodell, muss IL‑2 entweder exogen oder über IL-2-exprimierende Zellen, wie beispielsweise humane periphere mononukleäre Blutzellen (PBMC), zugegeben werden, um die Persistenz humaner Tregs in diesen Tieren über einen gewissen Zeitraum zu ermöglichen.

### Präklinische Tiermodelle

Präklinische pharmakologische und toxikologische Untersuchungen von genetisch modifizierten Zellen sind für deren klinische Entwicklung eine Grundvoraussetzung. Dieses setzt allerdings voraus, dass hierfür geeignete Tiermodelle vorhanden sind, was bei Therapiekonzepten mit genetisch modifizierten Lymphozyten aber eher die Ausnahme denn die Regel ist. Prinzipiell können hierfür zwar homologe Surrogatmodelle entwickelt werden, in denen anstelle der humanen Zellen beispielsweise möglichst vergleichbare murine Zellen in einem Mausmodell verwendet werden. Für den Nachweis des allgemeinen Wirkprinzips sind derartige homologe Modelle gut einsetzbar. Für die Überprüfung der Sicherheit dieser Zellen führt aber die Tatsache, dass nicht das eigentliche Zellprodukt, sondern ein Surrogat getestet wird, oftmals zu Unsicherheiten, inwieweit die damit erzeugten Daten am Ende auf den Menschen übertragbar sind. Ein homologes Modell ist daher immer sehr sorgfältig auf seine Relevanz für bestimmte Fragestellungen hin zu prüfen. Für pharmakologische Untersuchungen können Modelle herangezogen werden, in denen eine allogene Abstoßungsreaktion induziert wird, die bei Zugabe von genetisch modifizierten Tregs abgeschwächt ausfällt oder sich sogar verhindern lässt. Des Weiteren sind transgene Mauslinien beschrieben, die bestimmte humane HLA-Antigene, wie beispielsweise HLA-A2*02, exprimieren, und die damit als Tiermodelle für Toxizitätstests von TCR- oder CAR-modifizierten Tregs, die gegen dieses HLA-Antigen gerichtet sind, verwendet werden können [55]. Mithilfe dieses Modells lässt sich so etwa überprüfen, ob genetisch modifizierte Tregs unter gewissen Bedingungen nicht doch proinflammatorische Zytokine sezernieren und zu einem proinflammatorischen T‑Zellphänotyp hin transformieren. Auch die Aktivierung, Proliferation und Persistenz der TCR- und CAR-Tregs können in einem derartigen Modell adressiert werden. Grundsätzlich lässt sich aber festhalten, dass relevante Tiermodelle für genetisch modifizierte Tregs, wenn sie denn verfügbar sind, eher komplex und mit einigen Limitationen verbunden sind.

## Grundsätzliche klinische Aspekte

Für die klinische Entwicklung und Planung klinischer Prüfungen sind neben den allgemeinen rechtlichen Voraussetzungen ggf. vorhandene indikationsspezifische und produktspezifische Richtlinien bzw. Empfehlungen wissenschaftlicher Fachgesellschaften zu beachten [56].

Der Übergang von Arzneimittelkandidaten in die klinische Anwendung ist ein kritischer Schritt [57]. Die klinische Prüfung von T‑zellassoziierten Arzneimittelkandidaten hat gezeigt, dass klinisch relevante unerwünschte Arzneimittelwirkungen nicht immer in den vorangehenden nichtklinischen Studien identifiziert werden [58]. Relevante Tiermodelle, die solide Vorhersagen für erwünschte und unerwünschte Arzneimittelwirkungen erlauben, fehlen häufig im Kontext von CAR-T-Zellen [59]. Für Arzneimittelentwicklungen mit neuem Wirkmechanismus, ggf. langer Persistenz und nichtklinischen Daten in nur limitiert relevanten Modellen kann sich daher die Abschätzung eines hohen Risikos für den Übergang in die klinische Anwendung ergeben.

Häufigkeit und Schweregrad der Erkrankung sowie die (Nicht‑)Verfügbarkeit von Therapiealternativen werden die klinische Entwicklung beeinflussen [60–62]. Unter Berücksichtigung der Auswahl der geeignetsten Probanden- bzw. Patientenpopulation und risikominimierender Maßnahmen können auch Therapieansätze mit High-Risk-Arzneimitteln zu einer positiven individuellen Nutzen-Risiko-Bewertung und damit Genehmigung einer klinischen Prüfung führen. Zu den risikominimierenden Maßnahmen für erstmalige Anwendungen im Menschen gehören u. a. eine sichere Startdosis, eine gestaffelte Behandlung mit angemessenen Wartezeiten zwischen den ersten Studienteilnehmern sowie eine angemessen engmaschige Überwachung [63]. Bei (seltenen) schwerwiegenden Erkrankungen, die überwiegend oder nur Kinder betreffen, ist für den Übergang in die klinische Entwicklung die besondere Schutzbedürftigkeit der pädiatrischen Patientenpopulation zu berücksichtigen. Die Nachbeobachtungszeit sollte so gewählt werden, dass neben der gewünschten Persistenz der Wirkung auch die Langzeitsicherheit umfänglich adressiert wird.

Im Transplantationssetting kommen als Anwendungsgebiete für genetisch modifizierte T‑Zellen sowohl die Therapie der GvHD als auch die Verhinderung der Transplantatabstoßung in Betracht [11, 44]. Vorteile genetisch modifizierter Tregs könnten im Vergleich zu nativen (isolierten, ex vivo expandierten und reinfundierten) Tregs die verbesserte Antigenspezifität und -stabilität [64], die numerisch höhere Verfügbarkeit [36], und im Fall von Off-the-Shelf-Produkten zusätzlich die raschere Verfügbarkeit sein.

Sowohl für die GvHD als auch für die Verhinderung der Transplantatabstoßung gibt es Standardtherapien. Beim Übergang von der nichtklinischen in die klinische Entwicklung der genetisch modifizierten T‑Zellen ohne Nachweis von Sicherheit und Wirksamkeit werden die Tregs daher initial nur als Add-on-Therapie denkbar sein; eine etablierte wirksame Standardtherapie vorzuenthalten wäre ärztlich nicht vertretbar. Der Nachweis von Wirksamkeit der genetisch modifizierten Tregs unter Standardtherapie dagegen kann unter Umständen nicht erbracht werden. Endpunkte der klinischen Prüfung, Zeitpunkt der Gabe der genetisch modifizierten Tregs und ggf. Modifikation der Standardimmunsuppression müssen daher in der klinischen Prüfung gut begründet werden.

## Regulatorische Einordnung

Aus regulatorischer Sicht erfüllen genetisch modifizierte regulatorische T‑Zellen die Kriterien eines Arzneimittels für Neuartige Therapien (ATMP). Sie liegen in der Zuständigkeit des Paul-Ehrlich-Instituts (PEI) und unterliegen dem Arzneimittelgesetz (AMG). Die Verordnung über die Anwendung der Guten Klinischen Praxis bei der Durchführung von klinischen Prüfungen mit Arzneimitteln zur Anwendung am Menschen (GCP-V) gilt entsprechend. Durch die genetische Modifikation gelten Tregs in Deutschland auch als Arzneimittel, die aus einem gentechnisch veränderten Organismus (GVO) bestehen oder einen solchen enthalten. Bei Genehmigung einer klinischen Prüfung wird eine Freisetzungsgenehmigung für diesen GVO mitausgesprochen. Voraussetzung ist jedoch, dass im Zuge der Beantragung der klinischen Prüfung auch Unterlagen eingereicht werden, die eine Beurteilung des Umweltrisikos dieser Zellen erlauben. Da bei Freisetzung von genetisch modifizierten Zellen das Umweltrisiko in der Regel überschaubar ist, wird bei der Umweltrisikobewertung im Wesentlichen überprüft, ob ein Risiko durch die nichtvollständige Abreicherung von, für die genetische Modifikation eingesetzten, infektiösen viralen Vektorpartikeln und/oder durch eine Kontamination mit replikationskompetenten viralen Vektorpartikeln nicht ausreichend ausgeschlossen werden kann. Ein darauf fokussiertes und von Deutschland akzeptiertes Antragsformular, welches in mehreren EU-Mitgliedsstaaten verwendet werden kann, ist unter https://ec.europa.eu/health/human-use/advanced-therapies_en abrufbar.

## Fazit

Grundsätzlich muss aufgrund der breiten Spanne möglicher genetischer Manipulationen, deren Umsetzung und der klinischer Einsatzgebiete von genetisch modifizierten Tregs das Anforderungsprofil an einen klinischen Prüfungsantrag immer von Fall zu Fall beurteilt werden. Es ist jedoch zu erwarten, dass sich die regulatorische Sichtweise und Schwerpunktsetzung für die genetisch modifizierten Tregs in Analogie zu den CAR-exprimierenden Teff-Zellen für onkologische Indikationen mit den zunehmenden wissenschaftlichen Erfahrungen und Erkenntnissen weiterentwickeln werden.
